# Visco-Hyperelastic Model with Damage for Simulating Cyclic Thermoplastic Elastomers Behavior Applied to an Industrial Component

**DOI:** 10.3390/polym10060668

**Published:** 2018-06-15

**Authors:** Rafael Tobajas, Daniel Elduque, Elena Ibarz, Carlos Javierre, Alfonso F. Canteli, Luis Gracia

**Affiliations:** 1 Department of Mechanical Engineering, University of Zaragoza, C/María de Luna, 3, 50018 Zaragoza, Spain; rafaeltobajasalonso@gmail.com; 2 i+aitiip, Department of Mechanical Engineering, University of Zaragoza, C/María de Luna, 3, 50018 Zaragoza, Spain; carlos.javierre@unizar.es; 3 i3A, Department of Mechanical Engineering, University of Zaragoza, C/María de Luna, 3, 50018 Zaragoza, Spain; eibarz@unizar.es (E.I.); lugravi@unizar.es (L.G.); 4 Department of Construction and Manufacturing Engineering, University of Oviedo, C/Pedro Puig Adam, 33203 Gijón, Spain; afc@uniovi.es

**Keywords:** thermoplastic elastomers, visco-hyperelasticity, damage, cyclic uniaxial loading, mechanical characterization

## Abstract

In this work a nonlinear phenomenological visco-hyperelastic model including damage consideration is developed to simulate the behavior of Santoprene 101-73 material. This type of elastomeric material is widely used in the automotive and aeronautic sectors, as it has multiple advantages. However, there are still challenges in properly analyzing the mechanical phenomena that these materials exhibit. To simulate this kind of material a lot of theories have been exposed, but none of them have been endorsed unanimously. In this paper, a new model is presented based on the literature, and on experimental data. The test samples were extracted from an air intake duct component of an automotive engine. Inelastic phenomena such as hyperelasticity, viscoelasticity and damage are considered singularly in this model, thus modifying and improving some relevant models found in the literature. Optimization algorithms were used to find out the model parameter values that lead to the best fit of the experimental curves from the tests. An adequate fitting was obtained for the experimental results of a cyclic uniaxial loading of Santoprene 101-73.

## 1. Introduction

The use of thermoplastic elastomers has experienced an unprecedented increase in recent decades [1]. Several of the main reasons for that lie in their mechanical properties such as the ability to deform and vibration absorption capacity. In addition, lightness, manufacturing capacity, deformability and other advantages of these materials make them suitable for the manufacture of components helping to produce increasingly compact, lightweight and efficient vehicles [2]. Hence, they are among the most commonly used materials in sectors such as aeronautics and automotive [3].

Competitive industrial applications of thermoplastic elastomers must ensure the safe and durable design of mechanical and structural components. This involves the adequate mechanical characterization of these materials to be used subsequently in the development of numerical models, which in this way, are capable of simulating as accurately as possible their real behavior under a wide range of loading conditions [4,5,6].

Many constitutive models have been proposed in the literature [7,8,9] to simulate the mechanical behavior of thermoplastic elastomers under different loading conditions. The complex relationships existing among microstructure, strain, stress, temperature, etc. illustrates the difficulty of succeeding in choosing the adequate model [10].

Despite the new features of the constitutive models that have been published in recent years, models implemented in commercial codes only reproduce some partial aspects of the real mechanical behavior of these materials [8,11,12,13,14,15]. A reason for this may be due to the amorphous character of thermoplastic elastomer material microstructure, that generate a complex nonlinear response that depends on time, temperature, stress and strain history [9].

The hyperelastic and viscoelastic behaviors are characteristic of elastomers [16,17,18,19,20,21,22,23,24,25,26,27,28]. Since the relationship between stress and strain is nonlinear and time-dependent, it is necessary to resort to constitutive models able to reproduce both phenomena. Generally, the constitutive relations between stress and strain in elastomeric materials are expressed in terms of strain energy density (W or SED), which is a function of the material properties and the deformation experienced [22].

From the phenomenological hyperelastic models existing in the literature most of them can be classified among those defining the strain energy density as a scalar function of material properties and deformation invariants, and those using principal stretches instead [19,29].

A classical approach for simulating the viscoelastic behavior is represented by response analogy of certain mechanical components, such as springs and dampers [13,30], as for instance:

- Maxwell model consisting of two elements (spring and damper) in series [31,32].
- Spring and Maxwell model in parallel [33].
- Kelvin or Voigt model in which the two elements (spring and damper) are connected in parallel [34].
- Zener standard linear model obtained by adding a spring element in series to the Kelvin model [33].
- Burgers four element model obtained by combining Maxwell and Kelvin models in series [33].
- More complex models with multiple elements that combine the preceding more elementary models to reproduce real materials [35].

Another important mechanical feature exhibited by this type of materials is the damage effect. Two main types of damage theories exist, which are classified in micromechanical and phenomenological damage models, respectively [36]. Although both try to describe the same effect, several differences exist between them: micromechanical models allow micro-mechanisms to be captured explicitly by introducing internal variables such as dislocations, slips, etc., and hence are generally more accurate than phenomenological models. On the other side, phenomenological models generally simulate the material behavior on the macro-scale level using homogenized variables such as deformation gradient, velocity gradients, etc. They are simpler and suitable to analyze experimental results and to represent them in an analytical form but unsuitable beyond the domain in which they are defined [37]. However, it is difficult to incorporate variables such as the size effect or dislocations into phenomenological models through global parameters. Several micromechanical models of damage have been developed for this kind of materials [38,39,40,41].

In elastomeric materials, the damage effect is evidenced in phenomenological terms as the softening behavior during the first loading cycles after which the mechanical behavior stabilizes until it becomes repetitive. Though the first research on this effect was performed more than forty years ago by Mullins [42], no simple numerical model seems to be capable of representing the phenomenon accurately irrespective of the particular material experiencing this effect [11,43]. Throughout all the references, experimental evidence of this effect is observed, being usually remarkable in elastomers. Several authors have given a physical explanation of the phenomenon, and several theories have been exposed, but none of them has been unanimously endorsed [44].

This paper aims to formulate a phenomenological visco-hyperelastic, damage inducing model able of reproducing as accurately as possible the actual mechanical behavior of Santoprene 101-73 material. Account given of the complexity in the derivation of micromechanical damage models and its incorporation into phenomenological models, the size effect, dislocations, etc. were not considered in this work. Further, the damage effect has been simulated by Mullins theory. In this way, the presented model can be contemplated as the base of a constitutive material model that we will be able to implement in future works in some simulation commercial software such as Abaqus by an user-defined material model (UMAT) [13].

## 2. State of the Art Review

In this Section, the main constitutive material models used in the industry and the literature are revised to explain why they are used in this work. The revision advances from the simplest model to the more advanced model in such way that they can achieve the complex nonlinear response of the elastomeric materials [27].

### 2.1. Linear Elastic Model

Linear elastic model is maybe the constitutive material model most used in the industrial sector. Its ease to be understood and its simplicity make that to be used it is not necessary an advanced knowledge about material engineering. Another reason for that its use is so extended is that it ever converges in a result although this is not the more precise result. Hence, in terms of computation, for this model is not necessary to use an advanced software.

This constitutive model is usually used to describe materials that their stresses are proportional with their strains, they have not dependence on the rate of the loading or straining and, in addition, they return to their original shape when the loads are removed. The model for uniaxial behavior is characterized by a parameter called Young’s modulus *E* that represents the proportionality between stress *σ* and strain *ε* as:

(1) σ=E·ε

Although linear elastic model is widely used in industrial sector for rubber-like materials because of its simplicity and simulation rate, for academic and rigorous studies, it is not usually to use this constitutive material model to simulate elastomers. However, some studies can be found in the literature. [45]

### 2.2. Hyperelastic Models

To express the constitutive relations of the material under study, different hyperelastic models were considered, as for example: Neo-Hookean, Mooney-Rivlin, Yeoh or Ogden models [46,47,48,49,50].

One of the most used is the Ogden model thanks to its results. In this model, the strain energy density function is expressed as*:*

(2) $$W=W_{iso}\left(\lambda_{1},\lambda_{2},\lambda_{3}\right)+W_{vol}\left(J\right)=\overset{n}{\underset{i=1}{\sum }}\frac{2\mu_{i}}{\alpha_{i}^{2}}\left(\lambda_{1}^{\alpha_{i}}+\lambda_{2}^{\alpha_{i}}+\lambda_{3}^{\alpha_{i}}-3\right)+\overset{n}{\underset{i=1}{\sum }}\frac{1}{D_{i}}{\left(J-1\right)}^{2i}$$

where:

*W*: Strain energy density.

*μ_i_*, α_i_, *D_i_*: Model parameters.

*λ_i_*: Principal stretches.

*W_iso_*: Isochoric part of strain energy density function.

*W_vol_*: Volumetric part of strain energy density function.

*J*: Determinant of the deformation gradient tensor **F**.

It is assumed the incompressibility of the material. This assumption can be written as J−1=0 and then, $W_{vol}=0$.

The Cauchy stress tensor is obtained as shown in Equations (3) and (4).

(3) $$\sigma_{i}=\frac{\lambda_{i}}{\lambda_{1}\lambda_{2}\lambda_{3}}\frac{\partial W}{\partial \lambda_{i}}$$

(4) $$\sigma_{i}=\frac{\lambda_{i}}{\lambda_{1}\lambda_{2}\lambda_{3}}\left[\frac{2\mu_{i}\lambda_{i}^{\alpha_{1}-1}}{\alpha_{1}}+\frac{2\mu_{i}\lambda_{i}^{\alpha_{2}-1}}{\alpha_{2}}+\frac{2\mu_{i}\lambda_{i}^{\alpha_{3}-1}}{\alpha_{3}}\right]$$

The conditions of uniaxial testing and incompressible material are imposed by:

(5) $$\lambda_{2}=\lambda_{3}=\frac{1}{\sqrt{\lambda_{1}}}$$

These conditions are introduced into Equations (2) and (4) obtaining Equations (6) and (7), respectively.

(6) $$W=\frac{2\mu_{1}}{\alpha_{1}^{2}}\left[\lambda_{1}^{\alpha_{1}}+2{\left(\frac{1}{\sqrt{\lambda_{1}}}\right)}^{\alpha_{1}}-3\right]+\frac{2\mu_{2}}{\alpha_{2}^{2}}\left[\lambda_{1}^{\alpha_{2}}+2{\left(\frac{1}{\sqrt{\lambda_{1}}}\right)}^{\alpha_{2}}-3\right]+\frac{2\mu_{3}}{\alpha_{3}^{2}}\left[\lambda_{1}^{\alpha_{3}}+2{\left(\frac{1}{\sqrt{\lambda_{1}}}\right)}^{\alpha_{3}}-3\right]$$

(7) $$\begin{array}{ll} \sigma_{1}=\frac{\lambda_{1}}{\lambda_{1}\lambda_{2}\lambda_{3}}\frac{\partial W}{\partial \lambda_{1}}= & \lambda_{1}\cdot \frac{2\mu_{1}}{\alpha_{1}^{2}}\left[\alpha_{1}\lambda_{1}^{\alpha_{1}-1}-\frac{\alpha_{1}{\left(\frac{1}{\sqrt{\lambda_{1}}}\right)}^{\alpha_{1}-1}}{\lambda_{1}^{1.5}}\right]+\lambda_{1}\cdot \frac{2\mu_{2}}{\alpha_{2}^{2}}\left[\alpha_{2}\lambda_{1}^{\alpha_{2}-1}-\frac{\alpha_{2}{\left(\frac{1}{\sqrt{\lambda_{1}}}\right)}^{\alpha_{2}-1}}{\lambda_{1}^{1.5}}\right]\\ & +\lambda_{1}\cdot \frac{2\mu_{3}}{\alpha_{3}^{2}}\left[\alpha_{3}\lambda_{1}^{\alpha_{3}-1}-\frac{\alpha_{3}{\left(\frac{1}{\sqrt{\lambda_{1}}}\right)}^{\alpha_{3}-1}}{\lambda_{1}^{1.5}}\right] \end{array}$$

This kind of material models are widely used in the literature to simulate the mechanical behavior of rubber-like materials. Some studies where these models are used are reported in [51,52,53].

### 2.3. Visco-Hyperelastic Model

Here the development of the viscoelastic model is presented. According to Holzapfel [19] the strain energy density function for a viscoelastic material is given as:

(8) W(C,Γ1,…,Γn)=Wvol∞(J)+WIso∞(C¯)+∑α=1mΥα(C,¯Γα)C¯=J−23 C

where:

*W*: Strain energy density function.

*W_vol_*: Volumetric part of strain energy density function.

*W_iso_*: Isochoric part of strain energy density function.

*J*: Determinant of the deformation gradient tensor **F**.

***C***: Right Cauchy-Green Tensor.

**ϒ**: Power dissipation (responsible of the viscoelastic response).

Г*_α_*: Variable to characterize creep or relaxation behavior of the material.

*m*: number of viscous damping branches in the Holzapfel model.

The stress response of a viscoelastic material can be expressed as:

(9) $$S=2\frac{\partial \left(C,\Gamma_{1},\ldots ,\Gamma_{n}\right)}{\partial C}=S_{vol}^{\infty }+S_{iso}\;S_{iso}=S_{iso}^{\infty }+\overset{m}{\underset{\alpha =1}{\sum }}Q_{\alpha }\;Q_{\alpha }=-2\frac{\partial \Upsilon_{\alpha }\left(\bar{C},\Gamma_{\alpha }\right)}{\partial \Gamma_{\alpha }}$$

where:

***S****_vol_*: Volumetric response of the Piola-Kirchhoff second stress tensor.

***S****_iso_*: Isochoric response of the Piola-Kirchhoff second stress tensor.

**Figure 1:**
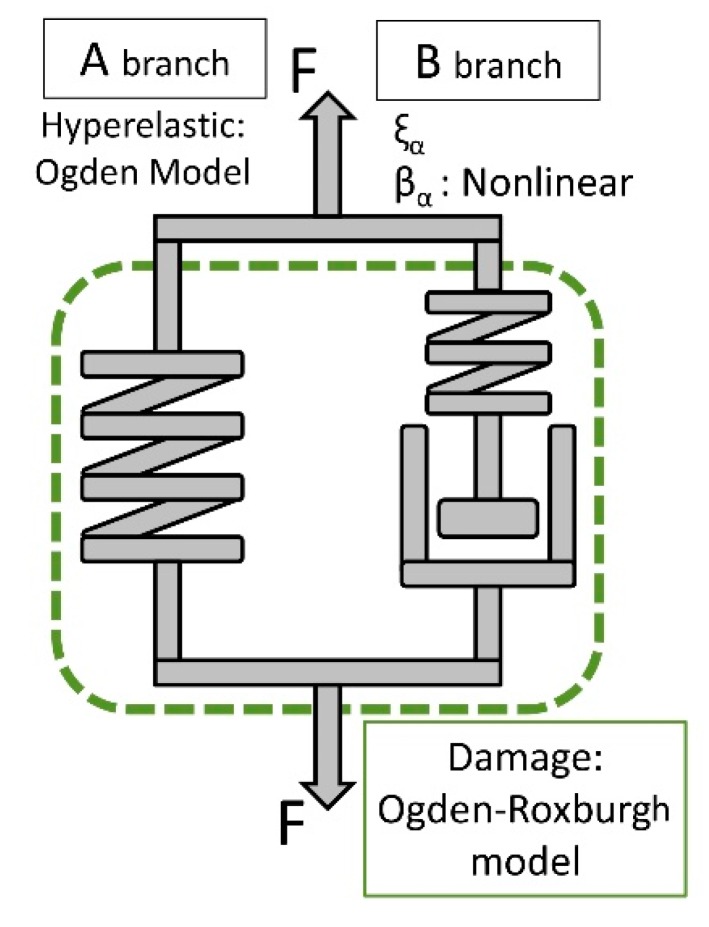
Rheological model representation.

*α*: number of Maxwell elements of the model. In this work *α* =1 (B branch of Figure 1).

*m*: number of viscous damping branches in the Holzapfel model.

***Q***: Isochoric non-equilibrium stress tensor.

And ***S*** is the Piola-Kirchhoff second stress tensor, which can be determined using a time integration algorithm:

(10) $$S_{n+1}={\left(S_{vol}^{\infty }+S_{iso}^{\infty }+\overset{m}{\underset{\alpha =1}{\sum }}Q_{\alpha }\right)}_{n+1}\;Q_{\alpha \left(n+1\right)}=\exp\left(2\zeta_{\alpha }\right)Q_{\alpha \left(n\right)}+\exp\left(\zeta_{\alpha }\right)\beta_{\alpha }\left(S_{iso\left(n+1\right)}^{\infty }-S_{iso\left(n\right)}^{\infty }\right)\;\zeta_{\alpha }=\frac{\Delta t}{2\tau_{\alpha }}$$

where

*β_α_*: Viscoelasticity coefficient to be determined.

*τ_α_*: Viscoelasticity coefficient to be determined. *m*: number of viscous damping branches in the Holzapfel model.

Δ*t*: Time increment.

*n + 1*: Actual step in the integration algorithm.

*n*: Previous step in the integration algorithm.

The visco-hyperelastic material models are the best models in the literature to simulate the mechanical behavior of elastomers. Nevertheless, their complex implementation and the convergence problems when they are used with the finite element method in no simple geometries, make that they are only used for academic purposes and they are not common for industrial sector. Some studies where these models are used are reported in [27,28,54].

### 2.4. Ogden-Roxburgh Damage Model

In this model, a scalar variable was introduced in the formulation of the strain energy density *W* [14]. Thus, the damage model is defined as a scalar function depending on the deformation gradient tensor ***F****:*

(11) W=W(F,η)W=ηW(F)+ρ(η)

where *η* is a scalar variable and *ρ*(*η*) is the damage function.

The *η* variable is continuous with respect to time, ranging from 0 to 1. During the phase in which the material does not undergo softening, the variable remains inactive taking the value 1. Otherwise, when softening appears, the variable becomes active and its values are calculated (0 ≤ *η* ≤ 1) allowing the damage effect to be characterized. If stresses are obtained by deriving the strain energy function, it occurs that stresses in the softening phase are the same as during the first deformation phase though multiplied by the scalar *η*. The scalar value as formulated by the Ogden-Roxburgh model is given by*:*

(12) $$\eta =1-\frac{1}{r}\mathrm{erf}\left(\frac{W_{max}-W}{m+\beta W_{max}}\right)$$

where:

erf: term to refer to the error function.

*r*: model parameter to be fitted.

*m*: model parameter to be fitted.

*β*: model parameter to be fitted.

*W_max_*: maximum value of strain energy density reached throughout the loading history.

*W*: strain energy density value of instantaneous and theoretical strain without damage.

## 3. Proposed Visco-Hyperelastic Model with Damage

In Section 1, a brief introduction about mechanical behavior of elastomers has been exposed. As it has been said, the complex response of this kind of materials depends mainly on the strain history, the strain rate and the internal damage effect experimented by the material. To take into account all these variables a new visco-hyperelastic material model is proposed.

The parameters *τ_α_* and *β_α_* of the viscoelastic model described in Section 2.3 exhibit constant values. In this section, a model is presented in which only a viscoelastic branch (*α* = 1) is considered. The “A” branch consists of a hyperelastic spring following the Ogden model whereas the “B” branch consists of a spring and a damper in series where the *β_α_* parameter is nonlinear. In the model described in Section 2.3 the *β_α_* parameter has a constant value. When the model was applied to the experimental trials it became clear that there was no precise fit. It was interpreted that there could be a dependence on the actual stiffness of the material for each level of load. It was therefore decided to include this dependency in the formulation of the model. Additionally, the Ogden-Roxburgh damage model is applied to both branches, according to Figure 1.

The second Piola-Kirchhoff stress tensor is computed by Equations (9) and (13).

(13) $$\beta_{\alpha }=\frac{\kappa \;\delta_{\alpha }\;\eta }{\left(\frac{\partial \sigma }{\partial \varepsilon }\right)}$$

where:

*∂σ*/*∂ε*: Tangent moduli of the hyperelastic model without damage.

*η*: Damage scalar parameter in Ogden-Roxburgh model.

*κ*: Viscoelasticity coefficient to be fitted.

*δ_α_*: Viscoelasticity coefficient to be fitted.

The value of *∂σ*/*∂ε* can be computed, for uniaxial tensile stress, by:

(14) $$\frac{\partial \sigma }{\partial \varepsilon }=\frac{\partial \sigma_{1}}{\partial \lambda_{1}}$$

where:

*σ*_1_: Computed by Equation (7)

*λ*_1_: Maximum principal stretch.

This model combines all inelastic effects that have been observed in the material experiments. The hyperelastic branch considers the non-linear behavior while the viscoelastic branch considers the strain rate. The reformulation of *βα* parameter allows the material behavior to be influenced by strain history and finally, all these effects are conditioned by the damage of the material during the strain cycles and that is characterized by the Mullins effect. In this work, a first experimental approach of the model is presented according to uniaxial experimental tests that are detailed below, and that are usually used in the industry to characterize these materials.

## 4. Experimental Tests

### 4.1. Material

**Figure 2:**
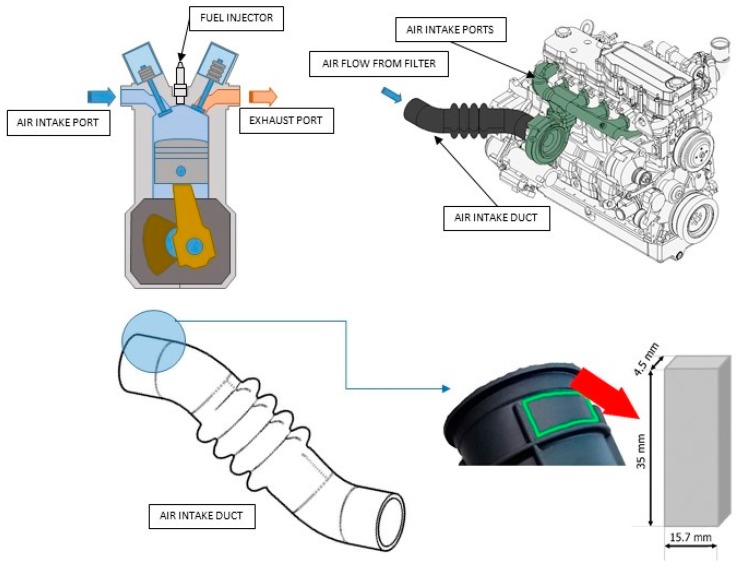
Santoprene 101-73 sample.

The material used in this work is Santoprene 101-73, manufactured by Exxon Mobil. This material is used to manufacture air intake ducts for a wide range of engines assembled in well-known brands of the automotive sector (Figure 2). According to the material manufacturer, it consists of a black and versatile thermoplastic vulcanizated (TPV) and a soft thermoplastic elastomer vulcanized (TPE) [55]. The material combines good physical properties and chemical resistance for its use in a wide range of applications.

Traditional TPEs are known as two-phase composites. Essentially, a hard thermoplastic phase is chemically or mechanically coupled with a soft elastomer phase resulting in a TPE with intermediate properties between the two phases [56].

This grade of Santoprene TPV can be processed by conventional thermoplastic equipment for injection molding, extrusion, or blow molding. Based on polyolefin, it is completely recyclable [55].

### 4.2. Experiments

To illustrate the visco-hyperelastic behavior and softening phenomenon of the Santoprene TPV, four cyclic uniaxial tensile tests were carried out on specimens extracted from an air duct automotive engine component. Such data are relevant because the mechanical behavior of the manufactured material, to be reproduced in this study, differs markedly from that exhibited by the virgin material as delivered by the manufacturer. This can be attributed to the manufacturing process implying notable temperature and pressure changes.

Taking into account the complex geometry of the duct component that impedes more suitable, conventional specimen shapes as “dumbbell” or “dogbone” ones [57,58,59], rectangular shaped specimens were used. The thickness, width, and length of the four specimens were 4.5 mm, 15.7 mm, and 35 mm, respectively, as shown in Figure 2.

**Figure 3:**
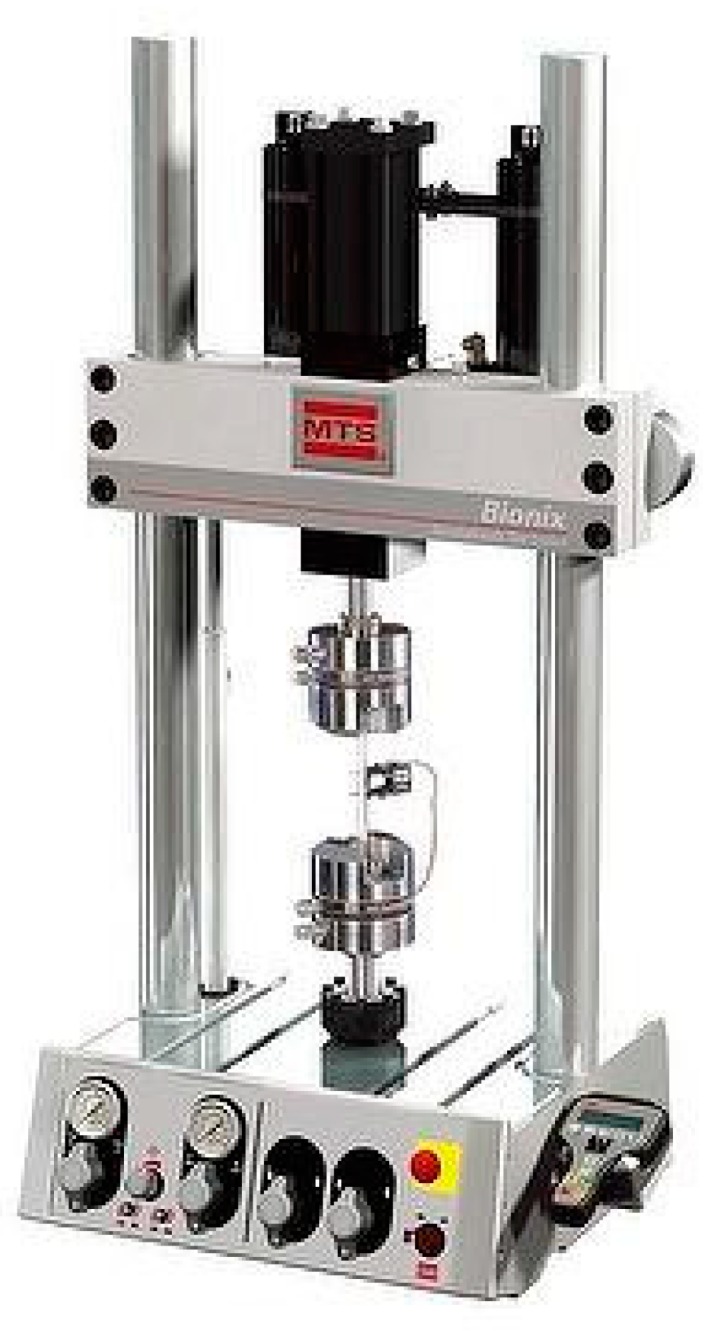
Bionix Servohydraulic Test System machine by MTS.

The uniaxial tests were performed in a dynamic testing machine “Bionix Servohydraulic Test System” manufactured by MTS [60] operating in displacement control mode (Figure 3). The specimens were subjected to uniaxial cyclic tests with maximum and minimum displacements according to Table 1 at a constant temperature of 23 °C up to 20,000 cycles (test frequency: 3 Hz).

**Figure 4:**
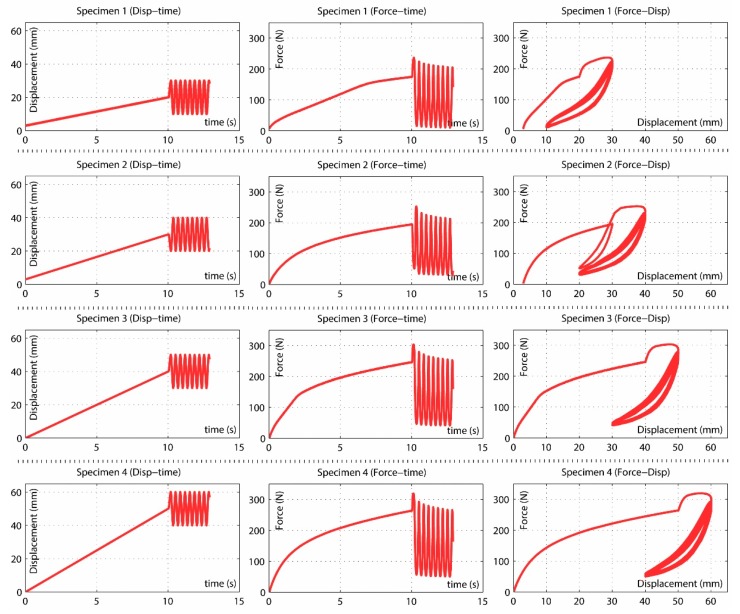
Force-displacement curves observed in the experiments.

The force and displacement were measured from data directly recorded by the testing machine. The force-displacement curves for the four specimens are presented in Figure 4, from which the following observations are drawn:

- The relation between stress and strain of the specimens in the first deformation cycle is non-linear.
- Before the maximum deformation is reached an abrupt change in the slope of the force-displacement curve is observed.
- The behavior is non-linear in subsequent cycles after the first deformation cycle.
- After the first deformation cycle, the force requested to induce the previous deformation happens to diminish notably.
- With increasing number of cycles, the applied force steadily reduces showing an asymptotic trend to a fixed value for a high number of applied cycles. Supposedly, this phenomenon can be attributed to the viscoelastic character of the material.
- Independently of the specimen tested, the force-displacement curve practically stabilizes after about 10,000 cycles.
- It follows that residual strains may induce specimens entering the plastic deformation regime.

In view of the above observations, we are able to state that the response of the Santoprene 101-73 is nonlinear and it depends on time, stress and strain history. Thus, the material behavior is driven by the hyperelastic behavior, viscoelastic behavior and Mullins effect. To reproduce the material response, the proposed numerical model, must accurately reproduce these three phenomena.

## 5. Fitting of Model Parameters from Experimental Data

Once the material has been characterized, all models explained in Section 2 have to be fitted with the experimental data to obtain the behavior parameters of this material [61]. The least-squares (LS) minimization method was used to identify the hyperelastic parameters in the present work. This minimization method is widely used to analyze and visualize data [62,63,64] and aims to find the minimum value of p minimizing the sum of squared errors (Equation (15)). The LS minimization method was programmed and solved in MATLAB [65].

(15) $$p=\overset{N}{\underset{i=1}{\sum }}{\left(Y_{\mathrm{experimental}}-Y_{\mathrm{predicted}}\right)}_{i}^{2}$$

where *N* is the number of points on the chart provided by the tests.

To generalize the proposed model, it is considered that the fitting parameters process could be done in a more general way by means of metaheuristic optimization algorithms, which are adequate for solving highly non-convex and nonlinear problems [66].

### 5.1. Elastic Model

The Young modulus for this model is fitted by using linear least squares minimization method. The value obtained is *E* = 3.27 MPa.

### 5.2. Hyperelastic Model

To set the hyperelastic model, the Ogden *N* = 3 formulation was chosen. After fitting the experimental data, the resulting non-dimensional model parameters are listed in Table 2.

### 5.3. Visco-Hyperelastic Model

After fitting the visco-hyperelastic model to the experimental data, the model parameters listed in Table 3 were obtained. They are obtained according to the fitting the model with the experimental results. However, they can be calibrated based on relaxation tests [67,68].

### 5.4. Ogden-Roxburgh Damage Model

To fit the damage model to the experimental data, the Roxburgh-Ogden formulation was adopted leading to the model parameters listed in Table 4.

To reproduce the force-displacement curves, Equations (1)–(12) were used by means of a numerical scheme based on displacement control as in the experimental tests for the same time step (0.01 s). The numerical scheme includes the following steps: calculation of principal stretches from test displacements values, calculation of strain energy density, parameters fitting for the hyperelastic model, calculation of stress tensor, calculation of Mullins parameter for damage and, finally, calculation of the resultant forces according to the specimen deformed dimensions. As the displacements are known at every time step, no prediction of displacements is needed, being possible to obtain all other quantities from the above equations. No problems of snapback or snapthrough were detected.

### 5.5. New proposed Model

As explained in Section 3, the proposed model includes a hyperelastic branch and a viscoelastic branch. The parameters used for hyperelastic branch are those specified in Table 2. It should be noted that for the viscoelastic branch the βα parameter depends on the strain history by the parameter ∂σ∂ε. The rest of viscoelastic branch parameter values are shown in Table 5. Finally, both branches of the model experience the Mullins effect by the same model and parameters specified in Section 5.4.

## 6. Results

Experimental tests shown in Figure 4 were simulated according to the previously presented models:

1. Linear elastic model as described in Section 2.1.
2. Hyperelastic behavior, applying the Ogden model as described in Section 2.2.
3. Visco-hyperelastic model as described in Section 2.2 and Section 2.3.
4. Visco-hyperelastic model with Ogden-Roxburgh damage model as described in Section 2.4.
5. Proposed visco-hyperelastic model with damage as described in Section 3.

**Figure 5:**
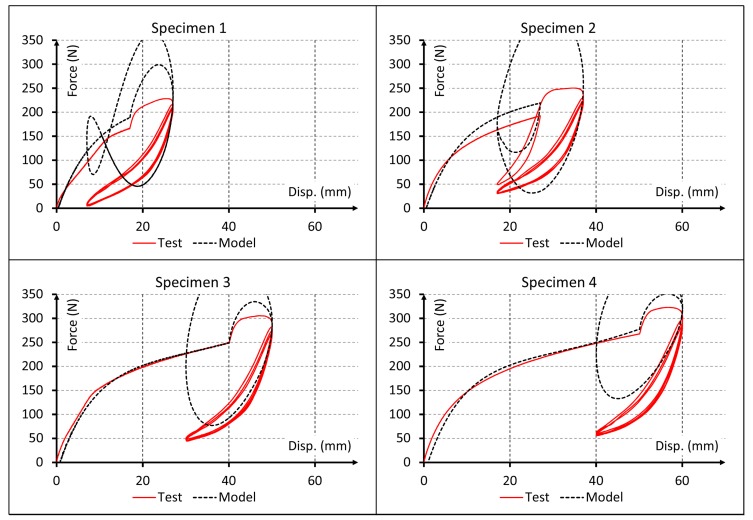
Comparison for first cycles between experimental data and force-displacement curves simulated using visco-hyperelastic model.

**Figure 6:**
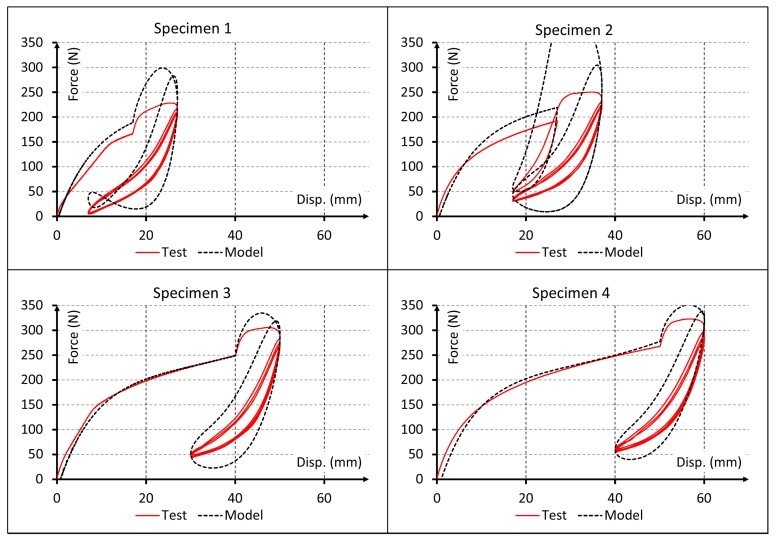
Comparison for first cycles between experimental data and force-displacement curves simulated using visco-hyperelastic model with Ogden-Roxburgh damage model.

**Figure 7:**
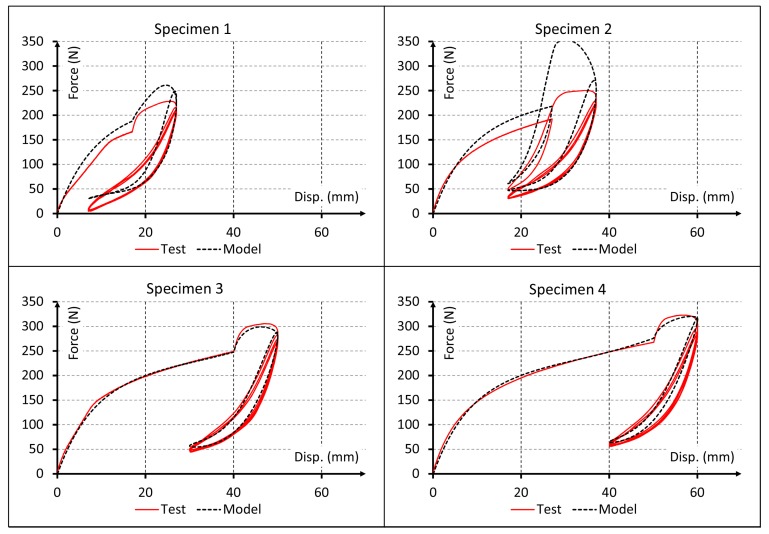
Comparison for first cycles between experimental data and force-displacement curves simulated using the proposed model.

**Figure 8:**
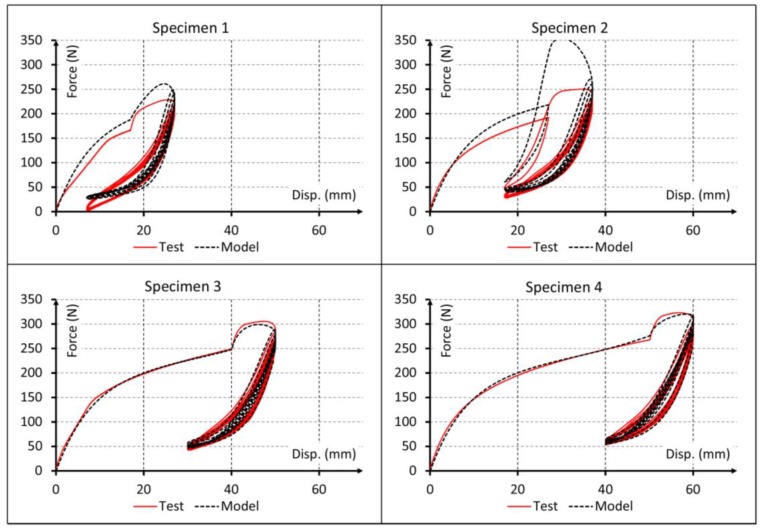
Comparison for all cycles between experimental data and force-displacement curves simulated using the proposed model.

Since the material behavior has clearly viscous effects, results for linear elastic and pure hyperelastic models are not plotted. Only the first cycles are represented into the plot results of Figure 5, Figure 6 and Figure 7 to obtain a proper comparison between experiments and simulations. In addition, a plot result for the proposed model and all cycles is shown in Figure 8.

Results for the visco-hyperelastic model are shown in Figure 5. In these plots the influence of viscous effects of the material can be observed but the rest of predictions are not as accurate as they could. Figure 6 shows the plot results for the visco-hyperelastic with Ogden-Roxburgh damage model explained in Section 2.4. The damage effect can be appreciated, and this improves the numerical results. These results are achieved applying models referenced in the literature, but they can be improved applying the new proposed model.

Finally, the results for the proposed model are shown in Figure 7 and Figure 8 and, visually, the numerical predictions are the best fit with test results. This is going to be checked in Section 7 using the coefficient of determination R^2^ for all the models.

## 7. Discussion

To be able to analyze better the results, the coefficient of determination R^2^ for all cases are obtained. In particular, Table 6 shows the values of this coefficient calculated for each model and experimental test, as well as the average values determined for each model.

A more detailed analysis of the simulation results indicates that Model 2 reproduces the nonlinear behavior of the material and Model 3 considers the viscous behavior of the material. Both can reproduce experimental data fairly well only for the first test cycle (Figure 5). While these models could capture the start point of subsequent cyclic loading rather precisely, they completely failed to reproduce the cyclic behavior of the material. Conversely, Model 4 could capture also the early stages of the cyclic behavior of material. This improvement in results can be visually observed in Figure 7 and Figure 8 and the data of Table 6 indicate that this model provides adequate results to simulate the mechanical behavior of elastomeric materials. Finally, Model 5 captured the whole cyclic behavior better than Model 4, especially for specimens 3 and 4 (Figure 7 and Figure 8), and the R^2^ values confirm that this model is the best of all.

The proposed visco-hyperelastic model with damage as described in Section 3 (i.e., Model 5 used in numerical simulations) allowed a satisfactory agreement to be achieved between theoretical predictions and experimental data. This is confirmed by Table 6. Since the R^2^ coefficient computed for Model 5 was by a large extent the nearest to 1, such a model must be considered the most suited for simulating the uniaxial behavior of the tested material.

## 8. Conclusions

In this work, a nonlinear phenomenological visco-hyperelastic model including damage was developed to simulate the uniaxial behavior of manufactured Santoprene 101-73. The main objective pursued in this investigation was to separate the different inelastic components (hyperelasticity, viscoelasticity and damage) influencing the material behavior, thus modifying/improving relevant former models of the literature. For that purpose, a nonlinear visco-hyperelastic model was derived based on the Ogden model with damage or softening (Mullins effect).

The formulation described above was utilized to simulate cyclic uniaxial loading. Specimens were extracted from an automotive duct component made of Santoprene 101-73 and submitted to cycling loading for different displacement ranges. Optimization algorithms were used to determine the model parameter values that allow the plotting of best fit force-displacement curves resulting from experimental tests. The proposed model fitted properly the whole loading history for specimens 3 and 4, showing better results that the models found in the literature.

It must be emphasized that the fitting of force-displacement curves was done only for available uniaxial tests data. Further, thermodynamic consistency of the model has not been considered. In future work, more experimental tests are necessary, both in configuration (i.e., biaxial, volumetric, and planar) and considering different strain rates, for the complete material characterization in case of complex stress states. Moreover, a more detailed damage formulation could be incorporated in future developments to predict the material stiffness loss at high number of load cycles.

## 9. Future Lines of Work

This study described the first stage in the development of a new constitutive model for rubber-like materials. Authors are working on the next steps for this model and its implementation in commercial finite element software. The complete model will be a huge advance in the design of this kind of materials because models such as the proposed one allow the studying of other effects of the material from the point of view of stresses. For example, fatigue phenomena are widely studied in metallic components from this point of view, but in rubber-like materials, these phenomena must be studied focusing on strains or energies. If a good constitutive material model is developed for this kind of material, maybe, the knowledge of metal fatigue can be used to improve the current elastomer fatigue models.

## Figures and Tables

**Table 1:** Displacements given to specimens in the experimental tests.

Samples	Displacements	
	Min. Displacement	Max. Displacement
Sample 1	10 (mm)	30 (mm)
Sample 2	20 (mm)	40 (mm)
Sample 3	30 (mm)	50 (mm)
Sample 4	40 (mm)	60 (mm)

**Table 2:** Ogden hyperelastic model parameter values fitted from experimental data.

Parameter	Value
μ_1_	11.515 (MPa)
α_1_	−1.979 (-)
μ_2_	19.930 (MPa)
α_2_	0.062 (-)
μ_3_	−27.425 (MPa)
α_3_	0.173 (-)

**Table 3:** Visco-hyperelastic model parameter values fitted from experimental data.

Parameter	Value
τ_α_	0.01 (s)
β_α_	−50 (-)

**Table 4:** Damage model parameter values fitted from experimental data.

Parameter	Value
*r*	1.338 (-)
*m*	0.236 (MPa)
β	0.116 (-)

**Table 5:** Proposed model parameter values fitted from experimental data.

Parameter	Value
τ_α_	0.01 (s)
δ_α_	−50 (-)
κ	0.7 (MPa)
$\frac{\partial \sigma }{\partial \varepsilon }$	Computed by Equation (14) (MPa)
η	Computed by Equation (12) (-)

**Table 6:** Values of *R*^2^ coefficient determined for each constitutive model and experimental test.

Models	Sample 1	Sample 2	Sample 3	Sample 4	Average
Elastic model	0.498	0.291	0.206	0.202	0.299
Hyperelastic model	0.539	0.371	0.331	0.316	0.389
Visco-hyperelastic model	0.548	0.400	0.347	0.348	0.411
Visco-hyperelastic with Ogden-Roxburgh damage model	0.889	0.860	0.876	0.915	0.885
Proposed model	0.972	0.979	0.986	0.976	0.978
